# Exercise Capacity and Clinical Outcomes in Chronic Heart Failure Patients with Mild Tricuspid Regurgitation

**DOI:** 10.3390/jcm12237459

**Published:** 2023-12-01

**Authors:** Kosuke Nakamura, Suguru Ishizaka, Kazunori Omote, Yutaro Yasui, Yoshifumi Mizuguchi, Sakae Takenaka, Yui Shimono, Ko Motoi, Hiroyuki Aoyagi, Yoji Tamaki, Sho Kazui, Yuki Takahashi, Kohei Saiin, Seiichiro Naito, Atsushi Tada, Yuta Kobayashi, Takuma Sato, Kiwamu Kamiya, Toshiyuki Nagai, Toshihisa Anzai

**Affiliations:** Department of Cardiovascular Medicine, Hokkaido University Graduate School of Medicine, Sapporo 060-8638, Hokkaido, Japan

**Keywords:** cardiopulmonary exercise testing, chronic heart failure, exercise intolerance, mild tricuspid regurgitation

## Abstract

Aim: The present study aimed to investigate the impact of mild tricuspid regurgitation (TR) on the exercise capacity or clinical outcomes in patients with chronic heart failure (CHF). Methods and Results: The study enrolled 511 patients with CHF who underwent cardiopulmonary exercise testing (CPET) between 2013 and 2018. The primary outcome was a composite of heart failure hospitalization and death. Patients with mild TR (*n* = 324) or significant TR (moderate or greater; *n* = 60) displayed worse NHYA class and reduced exercise capacity on CPET than those with non-TR (*n* = 127), but these were more severely impaired in patients with significant TR. A total of 90 patients experienced events over a median follow-up period of 3.3 (interquartile range 0.8–5.5) years. Patients with significant TR displayed a higher risk of events, while patients with mild TR had a 3.0-fold higher risk of events than patients with non-TR (hazard ratio (HR) 3.01; 95% confidence interval (CI), 1.50–6.07). Multivariate Cox regression analysis showed that, compared with non-TR, mild TR was associated with increased adverse events, even after adjustment for co-variates (HR 2.97; 95% CI, 1.35–6.55). Conclusions: TR severity was associated with worse symptoms, reduced exercise capacity, and poor clinical outcomes. Even patients with mild TR had worse clinical characteristics than those with non-TR.

## 1. Introduction

Tricuspid regurgitation (TR) is commonly observed in patients with cardiovascular disease (CVD) [1,2,3]. In patients with chronic heart failure (CHF), TR typically develops secondarily to right heart remodeling and dilation caused by elevated left ventricular (LV) filling pressure due to LV systolic or diastolic dysfunction, subsequent post-capillary pulmonary hypertension (PH), and pulmonary vascular remodeling (i.e., combined pre- and post-capillary PH (CpcPH)), which, in turn, leads to more severe right ventricular (RV) afterload [4,5].

Previous studies have reported that among patients with CVD, those with moderate and significant TR displayed worse symptoms and poorer clinical outcomes than those without significant TR [5,6,7,8,9,10,11]. Moreover, a recent study, using the National Echocardiography Database of Australia, demonstrated that even mild TR was independently associated with a significant increase in mortality in individuals who underwent echocardiography with or without CHF [12]. The majority of patients with CHF have mild TR rather than significant TR; nevertheless, the adverse effects of mild TR in patients with CHF are not well delineated.

We hypothesized that patients with mild TR have worse clinical characteristics, such as impaired functional capacity, and clinical outcomes than patients without TR. Therefore, investigating these clinical parameters may be of use in the risk stratification of patients with CHF. To test this hypothesis, we investigated the impact of mild TR on exercise intolerance and clinical outcomes in patients with CHF who underwent cardiopulmonary exercise testing (CPET).

## 2. Methods

### 2.1. Study Design

This was a single-center, observational, retrospective study that included consecutive patients whose exercise capacity was assessed by upright CPET using a ramp protocol between January 2013 and December 2018. CHF was defined as the evidence of heart failure (HF) based on the current European Society of Cardiology (ESC) diagnostic guideline [13] or previous HF-related hospitalization. All patients underwent CPET for assessing the exercise capacity were screened eligibility. Among the eligible patients, we excluded (1) patients without data of outcomes; (2) patients without CHF (defined above); and (3) patients with no echocardiography or B-type natriuretic peptide within 12 months from the day of CPET.

The study protocol was approved by the Ethics Committee of Hokkaido University Hospital (IRB No. 022-0048). The investigation conformed with the principles outlined in the Declaration of Helsinki. All the authors had full access to the data and take responsibility for its integrity.

### 2.2. Study Population

**Figure 1:**
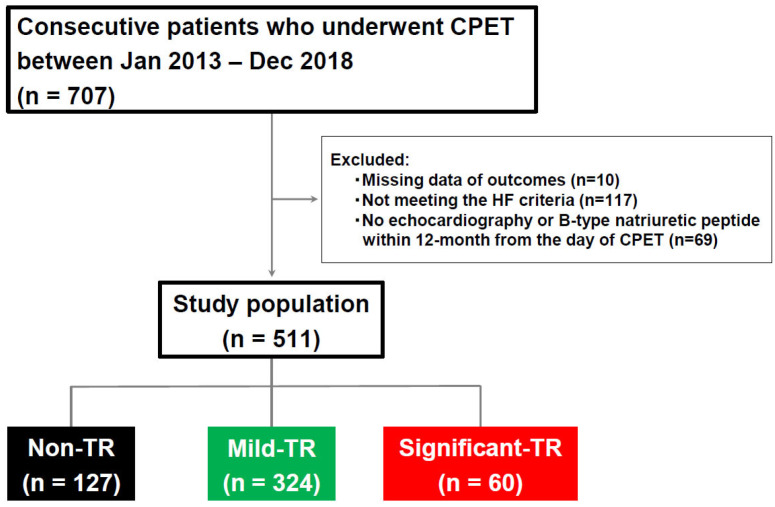
Flow diagram of the present study. Study population was divided into three groups by the severity of TR. CPET, cardiopulmonary exercise testing; HF, heart failure; TR, tricuspid regurgitation.

From the 707 consecutively enrolled patients who underwent CPET in this study, patients without outcome data (*n* = 10), echocardiography, or B-type natriuretic peptide (BNP) measurements within 12 months from the day of CPET (*n* = 69)or those who did not meet the HF criteria (*n* = 117) were excluded. Ultimately, 511 patients were examined. Patients were then divided into three groups according to the severity of TR: non-TR, mild TR, and significant TR (defined as moderate or severe TR) [14]. A study diagram of patient enrollment and grouping is shown in Figure 1.

### 2.3. Cardiopulmonary Exercise Testing

A symptom-limited standard upright cycle ergometry exercise using a cycle ergometer was performed, as described previously [15].

### 2.4. Cardiac Structure, Function, and Hemodynamics

Two-dimensional, M-mode, doppler, and tissue doppler echocardiography were performed according to the American Society of Echocardiography guidelines [16]. TR severity was classified as non (including trivial), mild, and significant (moderate or greater) according to the current recommendations for noninvasive evaluation of native valvular regurgitation [14]. The etiology of TR was defined as either primary or secondary. Primary TR was classified as documented congenital or organic causes of TR, and the others were classified as secondary TR [12].

The method of invasive hemodynamics assessment has been described previously [15].

### 2.5. Outcome Assessment

The primary outcome of interest was the composite of hospitalization due to worsening HF and death. The definition of death was all-cause death and that of hospitalization was hospitalization due to worsening heart failure. HF hospitalizations and mortality data were ascertained from medical records adjudicated by a single cardiologist (K.N.). Patient follow-up was initiated on the day of CPET.

### 2.6. Statistical Analyses

Continuous variables are presented as means ± standard deviations when normally distributed and as medians and interquartile ranges (IQRs) when non-normally distributed. Group differences were first compared using one-way ANOVA, the Kruskal–Wallis H test, or χ^2^ test, as appropriate. The Tukey honestl significant difference test or Steel–Dwass test was then used to compare between the individual groups. Event rates were compared using Kaplan–Meier curve analysis. Risk of the composite outcome was compared among the three patient groups according to the severity of TR (non-TR, mild TR, and significant TR). A Cox proportional hazards model was used to calculate the hazard ratio (HR) with a corresponding 95% confidence interval (95%CI), with adjustments for age, sex, moderate or greater mitral regurgitation (MR), and E/e’ ratio, which were the same co-variates used in a previous study [5].

A two-sided *p* value of <0.05 was considered statistically significant. All data were analyzed using JMP 16.0 (SAS Institute Inc., Cary, NC, USA) and Stata MP64 version 15 (StataCorp, College Station, TX, USA).

## 3. Results

### 3.1. Baseline Characteristics

**Figure 2:**
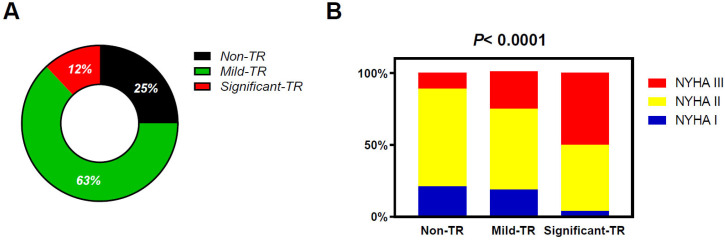
Distribution patterns of the TR severity in this study population (**A**) and the relation between the TR severity and NHYA functional class (**B**). TR, tricuspid regurgitation.

Overall, 25% of patients with CHF had non-TR, 63% had mild TR, and 12% had significant TR (Figure 2). Patients with TR were older with lower body mass index and had a higher prevalence of atrial fibrillation, moderate or greater MR, and poorer kidney function as compared with those with non-TR (Table 1). Patients with significant TR displayed lower prevalence of ischemic heart disease and significant aortic regurgitation as compared with other groups. There was no significant difference in terms of heart failure types classified by LVEF among the three groups. Patients with TR displayed higher plasma BNP levels, but patients with significant TR displayed higher BNP as compared to those with mild TR. Patients with significant TR displayed smaller LV diastolic dimension with larger LV mass index as compared to patients with non-TR or mild TR. Left atrial (LA) volume, RV dimension, and TR velocity were greater in patients with TR, but these abnormalities were the greatest in significant TR patients. Symptoms of HF assessed by NYHA functional class were worse in patients with mild TR than in patients with non-TR but were the worst in patients with significant TR (Figure 2).

### 3.2. Invasive Hemodynamics

Patients with significant TR displayed higher right atrial pressure (RAP) with higher pulmonary artery (PA) pressure as compared to those with non- or mild TR, while pulmonary capillary wedge pressure (PCWP) and pulmonary vascular resistance (PVR) were similar across the three groups. Patients with TR displayed more severe RV afterload, such as reduced pulmonary artery compliance (PAC) and elevated PA Ea as compared with those with non-TR. These were more severely impaired in patients with significant TR as compared to those with mild TR (Table 1).

### 3.3. Cardiopulmonary Exercise Testing

**Figure 3:**
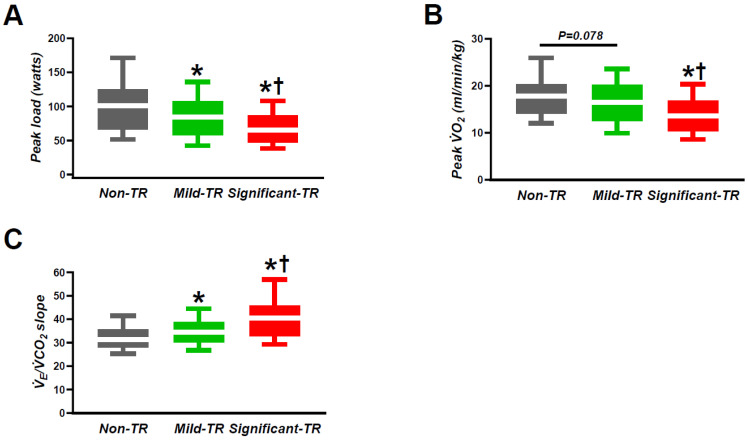
The relation between TR severity and the representative parameters in CPET: (**A**) peak load; (**B**) peak VO_2_; (**C**) V_E_/VCO_2_ slope. CPET, cardiopulmonary exercise testing; TR, tricuspid regurgitation; VCO_2_, carbon dioxide volume; VO_2,_ oxygen consumption; V_E_, minute ventilation. * *p* < 0.05 vs. non-TR. ^†^ *p* < 0.05 vs. mild TR.

Baseline heart rate was similar among the three groups, while peak heart rate and peak systolic blood pressure were lower in patients with significant TR as compared to those with the other two groups (Table 2). Patients with significant TR displayed lower peak oxygen consumption (VO_2_), anaerobic threshold, and O_2_ pulse compared to those with non- or mild TR. Patients with TR displayed higher minute ventilation (V_E_)/carbon dioxide volume (VCO_2_) slope and lower peak workload achievements as compared with those without TR, but these values were the most severely impaired in patients with significant TR (Figure 3, Table 2). Peak respiratory exchange ratio (RER) was similar across all groups and were all more than 1.10.

### 3.4. Tricuspid Regurgitation Severity and Clinical Outcomes

**Figure 4:**
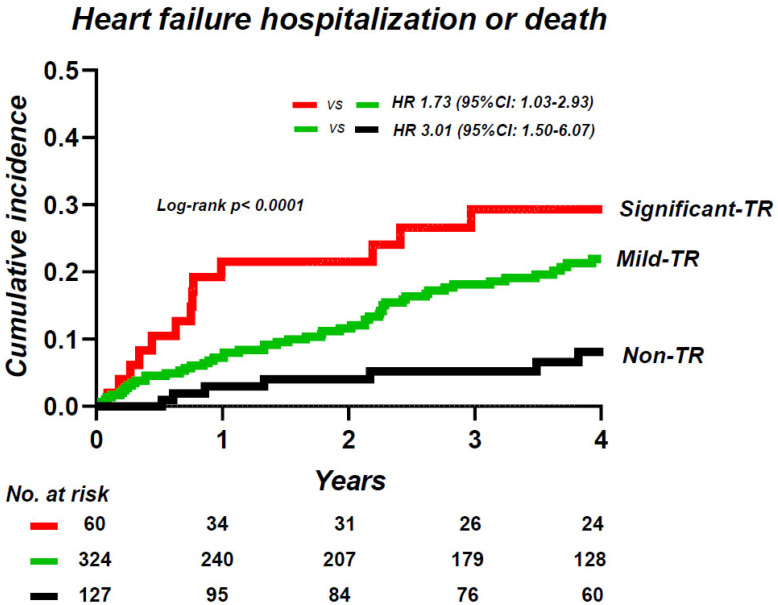
Kaplan–Meier analysis of composite of heart failure hospitalization and death, categorized by the severity of TR. TR, tricuspid regurgitation.

Over a median follow-up period of 3.3 (IQR 0.8–5.5) years, 90 patients experienced the composite endpoint, including 35 deaths (7%) and 67 HF hospitalizations (13%). Patients with significant TR displayed a higher risk of events compared to patients with non-TR (HR 5.22; 95% CI, 2.35–11.6) and to patients with mild TR (HR 1.73; 95% CI, 1.03–2.93). However, patients with mild TR showed a 3.0–fold higher risk of events as compared with those without TR (HR 3.01; 95% CI, 1.50–6.07) (Figure 4). In multivariable Cox regressions analyses, mild TR was associated with an increased risk of morbidity and mortality as compared with that of non-TR (HR 2.97; 95% CI, 1.35–6.55), while significant TR was associated with higher risks of adverse outcomes as compared to those with mild TR (HR 2.04; 95% CI, 1.13–3.67), even after adjusting for age, sex, moderate or greater MR, and E/e’ ratio, which were the same co-variates used in a previous study (Table 3) [5].

## 4. Discussion

The major finding of the present study was that the TR severity was associated with worse HF symptoms, reduced exercise capacity, and increased morbidity and mortality, and that in patients with CHF who underwent CPET, even patients with mild TR displayed worse clinical characteristics and outcomes than patients with non-TR. These findings suggest that the severity assessment of TR is useful for risk stratification in these patients.

### 4.1. Tricuspid Regurgitation in Chronic Heart Failure

In the early stages of HF, the LA compensates for LV dysfunction through its reservoir and booster functions as an important barrier between the LV and the lung [17]. However, as the disease progresses, dilation and dysfunction of LA advance over time. This progression leads to LA myopathy and is associated with post-capillary PH that increases RV afterload and leads to right-side heart dysfunction and the development of atrial and/or ventricle functional TR due to the dilatation of the annulus [3,4,5]. In the present study, patients with mild TR displayed greater LA volume and more severe pulsatile pulmonary vascular load (lower PAC and higher PA Ea) and greater RV dilation than those of non-TR patients. These parameters were more severely impaired in patients with significant TR than in the other groups.

### 4.2. Tricuspid Regurgitation and Functional Capacity

Many patients with TR are often asymptomatic [1], but in the advanced stages of the disease, patients display edema, anorexia, chronic liver or kidney congestion, or exertional fatigue [9,11]. Although the cause of impaired exercise capacity in TR patients is complex, a previous study that investigated the hemodynamics of exercise on right heart catheterization with a simultaneous expired gas analysis demonstrated that impaired exercise capacity (e.g., lower peak VO_2_) was related to an inability to adequately increase cardiac output (CO) response to exercise, coupled with elevated right and left heart filling pressures in patients with symptomatic grade 3 or 4 TR without significant left heart disease [6], wherein patients with mild TR were not emphasized. This impairment in perfusion was coupled with abnormal increases in left heart filling pressure that was related to elevated right heart pressure and enhanced diastolic ventricular interdependence (DVI) rather than a primary left heart lesion because LV transmural pressure (calculated as PCWP minus RAP) dropped with exercise, consistent with inadequate LV preload, despite elevated left-sided filling pressure [6,18,19]. When RV and pericardial pressures are low, LV end-diastolic pressure (LVEDP) reflects LV preload (LV end-diastolic volume (LVEDV)). However, in the presence of right heart enlargement and pericardial restraint, LVEDP and LVEDV become uncoupled [18,20,21]. This phenomenon is commonly observed in patients with significant TR and in those with persistent AF, obesity, RV dysfunction, or CpcPH [6,17,19,22]. Patients with these conditions demonstrate findings of exaggerated pericardial restraint and failure to augment CO, which may contribute to poor functional capacity. Similarly, in the present study, the LV ejection fraction was similar between all groups, but the RV dimension and LA volume were greater in patients with mild TR than in patients without TR, and these abnormalities were most prominent in significant TR patients. The enlargement of RV dimensions and LA volume leads to total heart enlargement that enhances the pericardial restraint and subsequent DVI. Therefore, these cardiac structural abnormalities may be related to a reduction in the augmented CO response to exercise, which further leads to reduced exercise capacity or worsening symptoms of HF.

### 4.3. Tricuspid Regurgitation and Clinical Outcomes in Chronic Heart Failure

Numerous studies have demonstrated that significant TR is associated with adverse outcomes in patients with CVD, such as HF with reduced EF, HF with preserved EF, left side valvular disease, or primary PAH [2,5,7,8,10]. However, the available data are scarce on the prognostic impact of mild TR in patients with CVD including CHF, despite a study from the National Echocardiography Database of Australia that first reported that even mild TR was independently associated with a significant increase in mortality in individuals with or without HF [12]. Herein, we showed that TR severity is independently associated with HF hospitalization or death, and that, compared with non-TR, even mild TR can increase the risk of adverse events, suggesting that TR severity may be useful for risk stratification in patients with CHF.

There are several limitations in this study. This is a single-center study. The study sample size was small, thereby hindering our ability to generalize the findings. Since the study population was enrolled between 2013 and 2018, the use of angiotensin receptor-neprilysin inhibitors or sodium-glucose cotransporter 2 inhibitors was limited.

## 5. Conclusions

TR severity was associated with exercise intolerance and adverse outcomes, where even mild TR was related to worse HF symptoms, reduced exercise capacity, and increased risk of morbidity and mortality in patients with CHF, suggesting that TR severity may be useful for risk stratification in these patients. Further study is required to identify therapeutic methods targeting TR to improve outcomes in patients with CHF.

## Figures and Tables

**Table 1:** Baseline vharacteristics.

	Non-TR (*n* = 127)	Mild TR (*n* = 324)	Significant TR (*n* = 60)	*p*-Value
Age (years)	53 ± 18	61 ± 15 *	60 ± 16 *	<0.0001
Women, *n* (%)	34 (27%)	101 (31%)	23 (38%)	0.28
Body mass index (kg/m^2^)	24.2 ± 4.9	22.8 ± 3.5 *	22.2 ± 4.0 *	0.014
Underlying heart disease				
DCM	29 (23%)	64 (20%)	12 (20%)	0.77
HCM	9 (7%)	36 (11%)	2 (3%)	0.07
HHD	13 (10%)	24 (7%)	2 (3%)	0.21
IHD	34 (27%)	72 (22%)	6 (10%) *^†^	0.02
Left side of valvular heart disease	21 (17%)	43 (13%)	8 (13%)	0.67
Others	21 (17%)	85 (26%)	30 (50%) *^†^	<0.0001
Comorbidities, *n* (%)				
Coronary disease	24 (20%)	64 (20%)	6 (10%)	0.20
Diabetes mellitus	29 (23%)	75 (23%)	13 (22%)	0.084
Hypertension	62 (49%)	137 (42%)	19 (32%)	0.97
Atrial fibrillation	15 (12%)	88 (27%)	26 (43%)	<0.0001
Medications, *n* (%)				
Renin-angiotensin system blocker	98 (77%)	252 (78%)	52 (41%)	0.098
Beta-Blocker	93 (73%)	254 (78%)	37 (62%)	0.019
Loop	52 (41%)	150 (46%)	45 (75%)	<0.0001
Laboratories				
Hemoglobin (g/dL)	13.7 ± 1.7	13.5 ± 4.9	12.9 ± 2.0 *	0.014
Estimated GFR (mL/min/1.73m^2^)	71 ± 27	64 ± 24 *	62 ± 24 *	0.004
BNP (pg/mL)	69.2 (37.7, 184)	126 (59.2, 246) *	146 (80, 402) *^†^	<0.0001
Echocardiography				
LV diastolic dimension (mm)	57 ± 10	57 ± 11	53 ± 13 *^†^	0.033
LV systolic dimension (mm)	44 ± 13	45 ± 15	41 ± 16	0.089
LV mass index (g/m^2^)	127 ± 49	124 ± 39	108 ± 42 *^†^	0.001
LA volume index (mL/m^2^)	40 ± 14	52 ± 23 *	68 ± 29 *^†^	<0.0001
LVEF (%)	44 ± 15	42 ± 16	43 ± 19	0.59
E/e’	10 (7, 12)	10 (8, 13)	10 (8, 15)	0.31
TAPSE (mm)	17 ± 5	16 ± 5	16 ± 5	0.18
RV basal diameter (mm)	39 ± 6	43 ± 6 *	49 ± 7 *^†^	<0.0001
RV mid-diameter (mm)	28 ± 4	32 ± 6 *	37 ± 7 *^†^	<0.0001
TR velocity (m/s)	2.3 ± 0.6	2.5 ± 0.4 *	3.2 ± 0.8 *^†^	<0.0001
Secondary TR	-	242 (75%)	39 (65%)	0.12
Moderate or greater mitral regurgitation	15 (12%)	78 (24%)	23 (38%)	0.0002
Moderate or greater aortic regurgitation	13 (10%)	24 (7%)	1 (2%) *^†^	0.04
Heart failure classification according to LVEF				0.36
HFrEF	62 (49%)	169 (52%)	28 (47%)	
HFmrEF	22 (17%)	54 (17%)	6 (10%)	
HFpEF	43 (34%)	101 (31%)	26 (43%)	
Invasive hemodynamics, *n* = 304				
Heart rate (bpm)	66 ± 13	67 ± 13	69 ± 15	0.69
Systolic BP (mm Hg)	123 ± 24	113 ± 23 *	108 ± 22 *	0.005
RAP (mm Hg)	7 ± 4	6 ± 3	10 ± 6 *^†^	0.040
PA systolic pressure (mm Hg)	30 ± 12	30 ± 11	42 ± 20 *^†^	0.0005
PA mean pressure (mm Hg)	20 ± 10	20 ± 8	26 ± 12 *^†^	0.002
PCWP (mm Hg)	11 ± 6	13 ± 7	14 ± 8	0.16
PVR (WU)	1.5 (1.2, 1.9)	1.7 (1.2, 2.4)	1.8 (1.3, 2.3)	0.078
PAC (mL/mm Hg)	5.4 ± 2.1	4.5 ± 2.3 *	3.0 ± 1.5 *^†^	<0.0001
PA Ea (mm Hg/mL)	0.4 ± 0.2	0.5 ± 0.2 *	0.7 ± 0.4 *^†^	<0.0001
CO (L/min)	4.8 ± 1.3	4.1 ± 1.1 *	4.0 ± 1.2 *	0.0003

Data are mean ± SD, median (interquartile range), or *n* (%). BNP, B-Type natriuretic peptide; BP blood pressure; CO, cardiac output; DCM, dilated cardiomyopathy; Ea, elastance, E/e’, ratio of early diastolic filling pulsed wave doppler to early diastolic mitral annular tissue doppler velocity; GFR, glomerular filtration rate; HCM, hypertrophic cardiomyopathy; HHD, hypertensive heart disease; HFrEF, heart failure with reduced EF; HFmrEF, heart failure with mildly reduced EF; HFpEF, heart failure with preserved EF; IHD, ischemic heart disease; LA, left atrial; LV, left ventricular; LVEF, left ventricular ejection fraction; PA, pulmonary artery; PAC, pulmonary artery compliance, PCWP, pulmonary capillary wedge pressure; PVR, pulmonary vascular resistance; RAP, right atrial pressure; RV, right ventricular; TAPSE, tricuspid annular plane systolic excursion; and TR, tricuspid regurgitation. * *p* < 0.05 vs. non-TR. ^†^ *p* < 0.05 vs. mild TR.

**Table 2:** Cardiopulmonary exercise testing.

	Non-TR (*n* = 127)	Mild TR (*n* = 324)	Significant TR (*n* = 60)	*p*-Value
Resting heart rate (bpm)	72 ± 13	70 ± 14	72 ± 15	0.37
Peak heart rate (bpm)	127 ± 29	119 ± 29	115 ± 30 *^†^	0.013
Resting systolic BP (mmHg)	118 ± 21	115 ± 21	108 ± 20 *	0.014
Peak systolic BP (mmHg)	166 ± 34	158 ± 36	142 ± 38 *^†^	<0.0001
Peak VO_2_ (mL/min/kg)	18.2 ± 5.7	16.9 ± 5.8	14.1 ± 4.9 *^†^	<0.0001
V_E_/VCO_2_	32.6 ± 6.6	35.0 ± 7.3 *	40.1 ± 10.5 *^†^	<0.0001
O_2_ pulse	9.8 ± 4.8	8.9 ± 3.2	7.3 ± 2.4 *^†^	<0.0001
Anaerobic threshold (ml/min/kg)	10.7 ± 2.9	10.4 ± 2.6	9.3 ± 2.6 *^†^	0.004
Peak load (Watts)	101 ± 43	88 ± 41 *	69 ± 33 *^†^	<0.0001
Peak RER	1.24 ± 0.14	1.24 ± 0.13	1.27 ± 0.14	0.42

Data are mean ± SD, median (interquartile range). BP, blood pressure; RER, respiratory exchange ratio; TR, tricuspid regurgitation; VCO_2_, carbon dioxide volume; VO_2_, oxygen consumption; V_E_, minute ventilation. * *p* < 0.05 vs. non-TR. ^†^ *p* < 0.05 vs. mild TR.

**Table 3:** Univariable and multivariable Cox proportional hazard model for composite events.

Variables	Univariable Analysis		Multivariable Analysis *	
	HR (95%CI)	*p*-Value	HR (95%CI)	*p*-Value
TR Mild vs. Non	3.01 (1.50–6.07)	0.002	2.97 (1.35–6.55)	0.007
TR Significant vs. Mild	1.73 (1.03–2.93)	0.040	2.04 (1.13–3.67)	0.018

CI, confidence interval; HR, hazard ratio; TR, tricuspid regurgitation. * The multivariable model was adjusted by age, sex, moderate or greater mitral regurgitation, and E/e’ ratio.

## Data Availability

Data sharing is not applicable to this article.
